# Stability analysis of genetic regulatory network with additive noises

**DOI:** 10.1186/1471-2164-9-S1-S21

**Published:** 2008-03-20

**Authors:** Yufang Jin, Merry Lindsey

**Affiliations:** 1 Department of Electrical and Computer Engineering, The University of Texas at San Antonio, San Antonio, TX, 78249, USA; 2 Division of Cardiology, Department of Medicine, The University of Texas Health Science Center at San Antonio, San Antonio, TX 78249, USA

## Abstract

**Background:**

Genetic regulatory networks (GRN) can be described by differential equations with SUM logic which has been found in many natural systems. Identification of the network components and transcriptional rates are critical to the output behavior of the system. Though transcriptional rates cannot be measured in vivo, biologists have shown that they are alterable through artificial factors in vitro.

**Results:**

This study presents the theoretical research work on a novel nonlinear control and stability analysis of genetic regulatory networks. The proposed control scheme can drive the genetic regulatory network to desired levels by adjusting transcriptional rates. Asymptotic stability proof is conducted with Lyapunov argument for both noise-free and additive noises cases. Computer simulation results show the effectiveness of the control design and robustness of the regulation scheme with additive noises.

**Conclusions:**

With the knowledge of interaction between transcriptional factors and gene products, the research results can be applied in the design of model-based experiments to regulate gene expression profiles.

## Background

Genetic networks regulate sophisticated biological functions by interacting genes and proteins and support homeostasis in metabolism and coordinate events during the developmental program. Research on stability analysis and regulation/control of these genetic networks are particularly important. Pioneer experimental studies in construction of genetic networks to manipulate protein levels or even to construct gene circuits with repressor functions have been carried out [1-9]. These experiments have demonstrated interesting properties of GRNs of *E. coli* in the presence of specified repressors. With different repressors, these GRNs include single or multiple interactions between genes and proteins. In a single gene regulatory network [1], the negative feedback that is integrated in the system decreases cell-to-cell fluctuations in protein concentration measurements. Distribution of the regulated protein concentration is proportional to the degradation rate of the gene network. In a two-gene regulation network [3], bi-stability is shown by coupling two proteins with negative regulation in the synthesis of each other. Stability analysis of this bi-gene regulation network is also presented based on parameter bifurcations. The significance of this experiment is that the transition between two stable states of the GRN is much sharper with respect to the intracellular stimuli, i.e., performance can be adjusted by special input. In a tri-gene regulatory model [2], three genes are regulated with cyclic repressibility. The system exhibited self-sustained oscillations over the entire growth phase of the host *E. coli* cells for certain biochemical parameters. These biological experimental results have shown that genetic networks can be regulated by a scheme of local promotor control, i.e. the number, type and placement of regulatory protein binding sites. However, quantitative analysis of the regulation function has not been studied.

The objective of our current study is to develop a mathematical model of the tri-gene regulation network and extend the theoretical stability analysis to the case with measurement noises. A novel control scheme is proposed to change the state of a genetic regulatory network by adjusting transcriptional rates. The proposed control scheme can provide biologists useful design techniques in model-based experiments to predict protein levels in genetic regulatory networks by adjusting specific regulatory factors. We will use the biological scheme of adjusting regulatory factors reported in research articles [9-14]. These regulatory factors will be adjusted based on errors between the measured gene products, mRNA and protein, concentration levels and their desired values to regulate the gene expressions profiles. The proposed control is based on the concentrations of *m*RNAs and proteins which can be easily measured by current molecular biology techniques. Therefore, it's useful when some of the transcription rates are unmeasurable.

We will introduce the general model of GRNs to explain the work, present the control design and the stability analysis , and then give a simulation example and conclusion.

## Mathematical model of genetic regulatory networks

**Figure 1:**
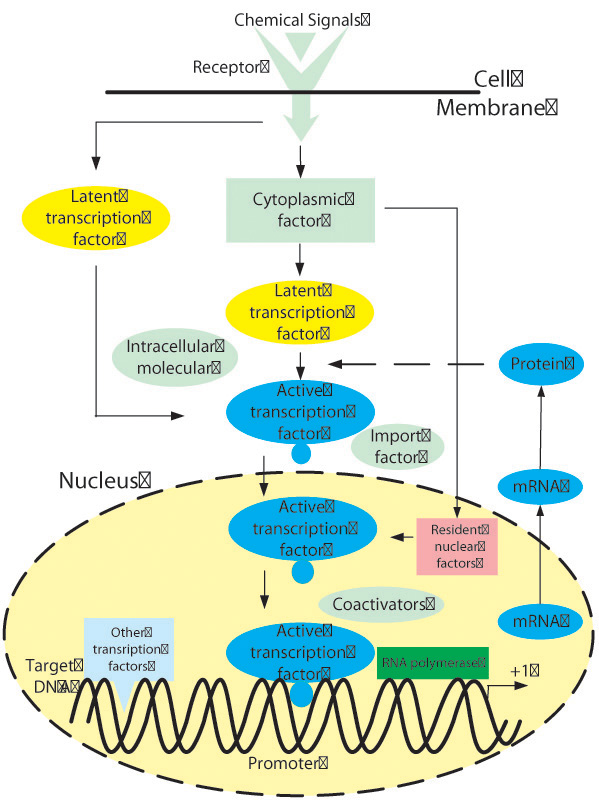
**A gene regulatory process**. A gene regulatory process includes input (chemical signals), output(proteins) and feedback (regulatory) factors.

A gene regulatory network for a eukaryote is shown in figure 1. Potential inputs of the system include a variety of developmental and environmental stimuli. System outputs are the synthesized proteins. Inputs activate a complex chain of intracellular reactions that activate a regulatory molecule, transcriptional factor, to translocate from the cytoplasm into the nucleus. In the nucleus, active transcription factors recognize a specific segment of DNA, termed a promotor. The promotor informs RNA polymerase, which binds closely to the promotor, where to start transcribing genetic information on DNA to mRNA. The mRNA molecules then leave the cell nucleus and enter the cytoplasm where proteins are synthesized in the presence of transfer RNAs. When the translated protein is capable of interacting with its own or other gene's transcription factor (denoted by the dashed arrow in figure 1, a regulatory or feedback loop is formed. Such transcriptional regulation is the typical method utilized by cells to control gene expression [9,15]. Feedback can occur in either a positive (activator) or a negative (repressor) direction. In a gene regulatory network, natural regulatory molecules are either activated transcription factors or proteins that activate transcription factors. Gene expression is very sensitive to changes in the composition of regulatory factors, which limits attempts to control gene expression. If artificial regulatory factors [10-14] can be used to change the activity of the transcription factors and the affinity of DNA binding, we can affect the transcriptional rate in GRNs and control the output behavior of the GRNs. Thus, in order to derive novel agents that regulate gene expressions, we must first understand GRN output behavior.

Two types of mathematical models have been developed to understand the working scheme of genetic networks: (1) Boolean networks and (2) sets of differential equations [9,16-19]. Boolean network model describes the expression of each gene with two states: ON or OFF, and the state of a gene is determined by a Boolean function of the states of other related genes. The differential equation model describes concentrations of mRNAs and proteins as continuous functions, which can provide more accurate and detailed dynamic information. Since genetic networks are high dimensional and nonlinear in nature, it is logical to consider such genetic networks from a nonlinear dynamic viewpoint, i.e., nonlinear differential equations. From a control point of view, main purpose of our mathematical model is to predict and manipulate the dynamic behaviors by analyzing available measurements. Researches of control design for Boolean networks have been carried out [20,21], while for continuous differential model, there are comparatively few literature references available [22].

In this section, the studied genetic network model is described by nonlinear differential equations

(1) $$\begin{array}{l} {\dot{m}}_{i}(t)=-k_{mi}m_{i}(t)+b_{i}(p_{1}(t),p_{2}(t),\;\;\;\ldots p_{m}(t))\\ {\dot{p}}_{j}(t)=-k_{pj}p_{i}(t)+k_{dji}m_{i}(t), \end{array}$$

where *m_i_*(*t*) and *p_j_*(*t*) are the concentrations of *m*RNAs and proteins in a genetic regulatory network, *i* = 1, 2, … , *n*, and *j* = 1, 2, … , *m*. Parameters *k_mi_* and *k_pj_* are degradation rates of the *m*RNAs and proteins, *k_dji_* are assumed to be constants describing the link between *m*RNAs and proteins. If the jth protein regulates the ith gene as illustrated by the dashed arrow in figure 1, there is a regulatory link in the network. Such regulation effects of proteins to gene expressions are described by wrapped nonlinear terms *b_i_*(*p*_1_(*t*), *p*_2_(*t*), … , *p_m_*(*t*)), which are nonlinear functions of *p*_1_(*t*), *p*_2_(*t*), … , *p_m_*(*t*), and can be described by SUM logic (additive effect) of each protein to the specified gene. Detailed description of the relationship between α_*i*_s, *b_i_*s and transcriptional factors have been previously published [9,17,18]. Adjustment of the regulatory factors will change parameters α_*ij*_ in the mathematical depiction written in equation (2).

(2) $$b_{ij}(p_{j}(t))=\left\{ \begin{array}{l} \alpha_{ij}\frac{{\left(p_{j}(t)/\beta \right)}^{H}}{1+{\left(p_{j}(t)/\beta \right)}^{H}}\text{ case 1 }\\ \alpha_{ij}-\alpha_{ij}\frac{{\left(p_{j}(t)/\beta \right)}^{H}}{1+{\left(p_{j}(t)/\beta \right)}^{H}}\text{ case 2} \end{array}\right.$$

where case 1 represents that transcription factor (protein) *j* is an activator of gene *i*, and in case 2, transcription factor *j* is a repressor of gene *i*. Parameters H in equation (2) is the Hill coefficient, β is a positive constant, and α_*ij*_ is the dimensionless transcription rate of transcription factor *j* to gene *i*. Equation (1) can be rewritten as the following form by applying (2)

(3) $$\begin{array}{l} \dot{m}(t)=Am(t)+Bg(p(t))+l_{0}\\ \dot{p}(t)=Cp(t)+Dm(t) \end{array}$$

where $m(t)=[m_{1}(t),\ldots m_{i}(t)\ldots m_{n}(t)]\in {ℜ}^{n},p(t)=[p_{1}(t),\ldots p_{j}(t)\ldots p_{m}(t)]\in {ℜ}^{m},A=diag\{-k_{m_{i}}\}\in {ℜ}^{n\times n},C=diag\{-k_{p_{j}}\}\in {ℜ}^{m\times m},D=diag\{k_{dji}\}\in {ℜ}^{m\times n}.$ Vector $l_{0}\in {ℜ}^{n}$ is determined by

(4) $$l_{0i}=\sum_{\Omega_{ij}}\alpha_{ij},$$

within Ω_*ij*_, which is a set of repressors for gene *i*. Matrix $B\in {ℜ}^{n\times m}$ describes the interconnection of transcription factors and genes in the network with element α_*ij*_,

$$B_{ij}=\{\begin{array}{ll} 0 & \text{no regulation for protein }j\text{ to gene }i,\\ \alpha_{ij} & \text{case 1,}\\ -\alpha_{ij} & \text{case 2,} \end{array}$$

and $g\left(p\left(t\right)\right)={\left[g\left(p_{1}\left(t\right)\right)\ldots g\left(p_{j}\left(t\right)\right)\ldots g\left(p_{n}\left(t\right)\right)\right]}^{T}\in {ℜ}^{m},g\left(p_{j}\left(t\right)\right)=\frac{{\left(p_{j}\left(t\right)/\beta \right)}^{H}}{1+{\left(p_{j}\left(t\right)/\beta \right)}^{H}}.$ Some special properties of the nonlinear function *g*(*p*(*t*)) should be pointed out:

• *g*(*p_j_*) > 0 always holds with *p_j_* > 0 and the equal sign holds only when *p_j_* =0;

• *g*(*p_j_*) is a monotonic increasing function, i.e. $\frac{\partial_{g}(p_{j})}{\partial p_{j}}\geq 0;$

• *g*(*p_j_*) satisfies a sector condition, which can be obtained from the mean-value theorem, $g\left(e_{j}+p_{j}\right)-g\left(p_{j}\right)=\frac{\partial g}{\partial p_{j}}|p_{j}=\xi \cdot e_{j},\xi \in \left[p_{j},p_{j}+e_{j}\right],$ and thus, $0\leq e_{j}[g\left(e_{j}+p_{j}\right)-g\left(p_{j}\right)]\leq \gamma_{j}e_{j}^{2},$ where $\gamma_{j}=\max\left\{\frac{H{\left(p_{j}/\beta \right)}^{H-1}}{\beta {\left(1+{\left(p_{j}/\beta \right)}^{H}\right)}^{2}}|p_{j}\in \left[p_{j},p_{j}+e\right]\right\}.$ The vector case of the above equation can be expressed as

(5) $$0\leq e^{T}\left[g\left(e+p\right)-g\left(p\right)\right]\leq \gamma e^{T}e,$$

where γ = max {γ_1_, γ_*j*_ …, γ_*m*_}.

Combine the state *m*(*t*), *p*(*t*) together, the state space model for control design and stability analysis is written as follows:

(6) $$\begin{array}{l} \dot{x}=f\left(t,x\right)=A_{c}x+B_{c}u+l,\;\;u=-g\left(y\right)\\ y=C_{c}x, \end{array}$$

where $x^{T}=\left[m^{T}\;p^{T}\right],l^{T}=\left[l_{0}^{T}\;0\right],\;x,\;l\in {ℜ}^{n+m},C_{c}=[0_{m\times n}\;I_{m\times m}]\in {ℜ}^{m\times (n+m)}$

$$A_{c}=\left[\begin{array}{cc} A & 0\\ D & C \end{array}\right]\in {ℜ}^{\left(n+m\right)\times \left(n+m\right)},\;\text{and}\;B_{c}=\left[\begin{array}{c} -B\\ 0 \end{array}\right]\in {ℜ}^{\left(n+m\right)\times n}.$$

## Methods: adaptive control of genetic networks in a noise-free case

Nonlinear adaptive control has been applied to many systems to improve performance. The control objective here is to drive the current state of genetic networks to desired values m^*^ and p^*^. In order to make the control design biologically meaningful, the following assumptions are introduced.

Assumption 1: Transcription rates of mRNAs in the studied genetic network are adjustable.

This assumption guarantees the possibility of adjusting the transcription rates and drive the current states to the desired values.

Assumption 2: State x^*T^ = [m^*T^ p^*T^] is the stable steady state generated by the same genetic network in equation (3) with desired transcription rates.

This assumption guarantees that m^*^ and p^*^ are achievable and has biological meaning for a real genetic network. The stability also guarantees that once the state is driven to x^*^, it will stay there.

The control design proposed here includes two parts: i) stability analysis of x^*^ generated by the genetic network with the desired degradation, transcription rate, the given sector parameter γ; ii) control design to drive the current states to the desired x^*^. Assume x^*^ is generated by

(7) $$\begin{array}{l} \dot{x}=A_{c}x+B_{c}^{*}u+l,\;u=-g\left(y\right),\\ y=C_{c}x. \end{array}$$

where $B_{c}^{*}$ is the desired transcription rates. Stability of the genetic network means: (1) State x^*^ is the equilibrium of equation (7), i.e. $A_{c}x^{*}-B_{c}^{*}g\left(C_{c}x^{*}\right)+l=0;$ (2) Starting at any initial states *x*_0_ close to x^*^, the trajectory will converge to x^*^ as time goes to infinity. If we define e^*^ = *x* − x^*^, the error *e* → 0 as *t* → ∞.

*Lemma 1: The system (7) is globally exponentially stable if the transfer function matrix G*(*s*) = *C_c_*(*sI* − *A_C_*)^−1^$B_{c}^{*}$is strictly positive real, i.e. with the controllable and observable pair$\left\{A_{c},B_{c}^{*},C_{c}\right\}$, *there exist positive matrices P and Q, P = P^T^, such that*

(8) $$PA_{c}+A_{c}^{T}P=-Q,\;PB_{c}^{*}=C_{c}^{T}.$$

*Proof:* With constant x^*^, the error dynamics can be expressed as

(9) $$\begin{array}{c} \dot{e}=A_{c}x+B_{c}^{*}u+l=A_{c}\left(e+x^{*}\right)-B_{c}^{*}g\left[C_{c}\left(e+x^{*}\right)\right]+l\\ =A_{c}e-B_{c}^{*}\left[g\left(C_{c}e+C_{c}x^{*}\right)-g\left(C_{c}x^{*}\right)\right]. \end{array}$$

Choosing the Lyapunov function $V_{1}(e)=\frac{1}{2}e^{T}Pe,$ its time derivative ${\dot{V}}_{1}(e)$ is obtained by the following equations.

(10) $$\begin{array}{c} {\dot{V}}_{1}=\frac{1}{2}\left({\dot{e}}^{T}Pe+e^{T}P\dot{e}\right)\\ =\frac{1}{2}\left\{e^{T}A_{c}^{T}-{\left[g\left(C_{c}e+C_{c}x^{*}\right)-g\left(C_{c}x^{*}\right)\right]}^{T}B_{c}^{*T}\right\}Pe\\ \;+\frac{1}{2}e^{T}p\left\{A_{c}e-B_{c}^{*}\left[g\left(C_{c}e+C_{c}x^{*}\right)-g\left(C_{c}x^{*}\right)\right]\right\}\\ =\frac{1}{2}e^{T}\left[PA_{c}+A_{c}^{T}P\right]e-e^{T}PB_{c}^{*}\left\{g\left[C_{c}e+C_{c}x^{*}\right]-g\left(C_{c}x^{*}\right)\right\}\\ =-\frac{1}{2}e^{T}Qe-{\left(C_{c}e\right)}^{T}\left\{g\left[C_{c}e+C_{c}x^{*}\right]-g\left(C_{c}x^{*}\right)\right\}\leq -\frac{1}{2}e^{T}Qe \end{array}$$

which is negative definite since (*C_c_e*)^*T*^ {*g*[*C_c_e* + C_c_x^*^)]—*g*(C_c_x^*^)} > 0 by applying equation (5). Thus, the error dynamics will converge to zero exponentially by satisfying the strict positive real condition given as equation (8) on the system parameters.

*Remark 1:* Lemma 1 gives a sufficient condition on parameter settings for a stable genetic network. The stability is determined by system parameters and the sector condition of the nonlinear feedback function.

Lemma 1 provides an easy way to check the stability of a genetic network, since it is easy to get the transfer function for linear time invariant system and check whether the transfer function is strictly positive real or not.

*Remark 2:* While applying the small gain theorem, with the consideration of the sector parameter γ, we can get the sufficient and necessary condition: $\sup_{\omega }|G\left(j\omega \right)|<1/\gamma ,\omega \in ℜ.$ Since the necessary condition is not involved in the following control design, we ignore the proof here. Related information of it can be found in [23-25].

Based on the above stability analysis, the following theorem gives the control design that drives the current state to the desired state by adjusting the transcription rates of the system.

*Theorem 1:* Assume transcription rates of gene *i* in system (7) can be adjusted by the control law

(11) $${\dot{\alpha }}_{ij}=-k_{ij}\sum_{i,j,k=1}^{i=n,j=m,k=n+m}e_{k}P_{ki}g_{j},$$

where *i* = 1, 2, … , *n, j* = 1, 2, … , *m*, *e_k_* is the *k*th element in the error vector between the current state and the desired state, i.e., *e* = *x*—x^*^, *g_j_* = *g*(*p_j_*(*t*)), *P_ij_* is the element in the positive definite matrix P defined in equation (8). The system (6) will converge to the desired state x^*^ asymptotically as time goes to infinity.

Proof: From equations (6, 7), the error dynamics is

(12) $$\begin{array}{c} \dot{e}=A_{c}x+B_{c}u+l=A_{c}e-B_{c}g\left(y\right)+B_{c}^{*}g\left(y*\right)\\ =A_{c}e-\left(B_{c}-B_{c}^{*}\right)g\left(y\right)-B_{c}^{*}\left[g\left(y\right)-g\left(y*\right)\right] \end{array}$$

Choose Lyapunov candidate $V\left(e,{\tilde{\alpha }}_{ij}\right)=\frac{1}{2}e^{T}Pe+\sum_{i,j=1}^{i=n,\;j=m}\frac{1}{2k_{ij}}{\tilde{\alpha }}_{ij}^{2},$ where ${\tilde{\alpha }}_{ij}\text{s}$ are defined as ${\tilde{\alpha }}_{ij}=B_{c}-B_{c}^{*}=\alpha_{ij}-\alpha_{ij}^{*}.$ Recall that the bottom block with *m* × *n* dimension in both matrices are zeroes, ${\tilde{\alpha }}_{ij}$ is only related to the transcription rates in the system. Thus, the time derivative of the Lyapunov function can be derived as follows.

(13) $$\begin{array}{c} \dot{V}=\frac{1}{2}\left({\dot{e}}^{T}Pe+e^{T}P\dot{e}\right)+\sum_{i,j=1}^{i=n,\;j=m}\frac{1}{k_{ij}}{\tilde{\alpha }}_{ij}{\dot{\tilde{\alpha }}}_{ij}\\ =\frac{1}{2}\left\{e^{T}A_{c}^{T}-g{\left(y\right)}^{T}{\left(B_{c}-B_{c}^{*}\right)}^{T}-{\left[g\left(y\right)-g\left(y*\right)\right]}^{T}B_{c}^{*T}\right\}Pe\\ +\frac{1}{2}e^{T}P\left\{A_{c}e-\left(B_{c}-B_{c}^{*}\right)g\left(y\right)-B_{c}^{*}\left[g\left(y\right)-g\left(y*\right)\right]\right\}+\sum_{i,j=1}^{i=n,\;j=m}\frac{1}{k_{ij}}{\tilde{\alpha }}_{ij}\dot{\tilde{\alpha }}\\ =\frac{1}{2}e^{T}\left[PA_{c}+A_{c}^{T}P\right]e-e^{T}PB_{c}^{*}\left\{g\left[C_{c}e+C_{c}x^{*}\right]-g\left(C_{c}x^{*}\right)\right\}\\ -e^{T}P\left(B_{c}-B_{c}^{*}\right)g\left(y\right)+\sum_{i,j=1}^{i=n,\;j=m}\frac{1}{2k_{ij}}{\tilde{\alpha }}_{ij}{\dot{\tilde{\alpha }}}_{ij}\\ \leq -\frac{1}{2}e^{T}Qe-\sum_{i,j,k=1}^{i=n,j=m,k=n+m}e_{k}P_{ki}{\tilde{\alpha }}_{ij}g_{j}+\sum_{i,j=1}^{i=n,\;j=m}\frac{1}{k_{ij}}{\tilde{\alpha }}_{ij}{\dot{\tilde{\alpha }}}_{ij}\\ \leq -\frac{1}{2}e^{T}Qe \end{array}$$

with the adaption control chosen as ${\dot{\tilde{\alpha }}}_{ij}=-{\dot{\alpha }}_{ij}=k_{ij}\sum_{i,j,k=1}^{i=n,j=m,k=n+m}e_{k}P_{ki}g_{j}.$

From the above Lyapunov argument, with a positive definite *V* and negative definite $\dot{V}$ all the errors decrease to zero. This concludes the proof that the tracking error of the genetic network from current state to the desired state is globally asymptotically stable with the adaptive control given in (11), i.e., the states are driven to desired levels. As the tracking errors converge to zero, the adjustable transcription rates α_*ij*_ will converge to constants.

### Boundedness with additive noises

When signals are sensed, signal distortion, transmission delay and noise are unavoidable. In this section an additive measurement noise will be considered, and the distorted measurements of mRNA and protein concentration levels will be used in the adaption law.

Considering the aforementioned system in equation (6) with additive noises

(14) $$\dot{x}=f\left(t,x\right)+d\left(t,x\right),$$

where $f:{ℜ}^{+}\times {ℜ}^{n}\to {ℜ}^{n},d:{ℜ}^{+}\times {ℜ}^{n}\to {ℜ}^{n}$ are piecewise continuous in *t*, the systems can be viewed as the nominal system

(15) $$\dot{x}=f\left(t,x\right),$$

with perturbation term *d(t, x).* The variable *u* is a function of *x* and is omitted here for simplicity.

Assume the nominal system has an equilibrium point at the origin, if *d(t, x)* = 0 as *x* = 0, then the origin is still an equilibrium point with the disturbance *d(t, x).* However, in most cases, the disturbance does not satisfy this assumption, i.e. *d*(*t*, 0) ≠ 0. In this case, the origin is not an equilibrium point and no conclusion can be drawn about stability of the origin. The following Theorem shows that the best result we can expect is the uniform boundedness of the disturbed system when the origin is exponentially stable.

*Theorem 2:* Consider the system and adaptation laws given by equations (6), and (11), the system is uniformly bounded with disturbance ‖d(t,x)‖≤δ where δ is a positive constant. The upper bound *b_u_* and lower bound *b_l_* of the system is determined by

$$b_{u}=\frac{\alpha_{4}\delta }{\alpha_{3}\mu }\sqrt{\frac{\alpha_{2}}{\alpha_{1}}},\;\text{and}\;b_{l}=\frac{\alpha_{4}\delta }{\alpha_{3}\mu },$$

where 0 < μ < 1, respectively. α_*i*_s are positive constants defined as follows.

*proof* From the proof of Theorem 1, the error dynamics of the whole system is exponentially stable at the origin. Thus, there exist a Lyapunov function $V(\Phi ),\;\Phi ={\left[e,{\tilde{\alpha }}_{ij}\right]}^{T}$ satisfies the following condition:

(16) $$\begin{array}{l} \alpha_{1}{\left\|\Phi \right\|}^{2}\leq V\left(\Phi \right)\leq \alpha_{2}{\left\|\Phi \right\|}^{2},\\ \frac{\partial V}{\partial t}+\frac{\partial V}{\partial \Phi }f\left(t,\Phi \right)\leq -\alpha_{3}{\left\|\Phi \right\|}^{2},\\ \frac{\partial V}{\partial \Phi }\leq \alpha_{4}\left\|\Phi \right\|. \end{array}$$

With the additive noise disturbance (*t*, Φ) considered, the time derivative of *V*_2_ is rewritten as

(17) $$\begin{array}{c} \dot{V}\leq -\alpha_{3}{\left\|\Phi \right\|}^{2}+\alpha_{4}\left\|\Phi \right\|\cdot \left\|\left(t,\Phi \right)\right\|\\ \leq -\alpha_{3}{\left\|\Phi \right\|}^{2}+\alpha_{4}\delta \left\|\Phi \right\|\\ \leq -\alpha_{3}(1-\mu ){\left\|\Phi \right\|}^{2}-\alpha_{3}\mu {\left\|\Phi \right\|}^{2}+\alpha_{4}\delta \left\|\Phi \right\|\\ \leq -\alpha_{3}(1-\mu ){\left\|\Phi \right\|}^{2}, \end{array}$$

for any $\left\|\Phi \right\|\geq \frac{\delta \alpha_{4}}{\mu \alpha_{3}}.$ This gives the lower bound as $bl=\frac{\delta \alpha_{4}}{\mu \alpha_{3}}.$ From BIBO Theorem from the reference [26], the upper bound can be determined as $bu=\frac{\alpha_{4}\delta }{\alpha_{3}\mu }\sqrt{\frac{\alpha_{2}}{\alpha_{1}}}.$

From theorem 2, it can be shown that with bounded noises, both the state estimation errors and adaptation control are bounded. With disturbance bound δ → 0, differences between the adaptation control and the desired constant transcriptional rates shrink to zero also.

## Simulation results and discussion

**Figure 2:**
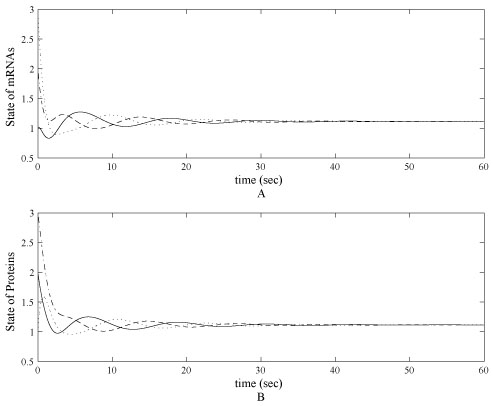
**Uncontrolled State of mRNA and protein levels**. (A) Uncontrolled state of mRNAs of *lacl, tetR, cl* in the tri-gene regulatory in a noise-free case. The initial states of computer simulation start from initial status as [3, 2, 1]. (B) Uncontrolled proteins *cl, lacl, tetR* in the tri-gene regulatory in a noise-free case. All states converge to 1.115 with in 50 second simulation time.

To show the effectiveness of the proposed control, an example is simulated with Matlab. We consider the dynamics of the tri-gene network described in [2].

(18) $$\begin{array}{c} {\dot{m}}_{i}(t)=-k_{mi}m_{i}+\frac{\alpha_{i}}{1+p_{j}^{n}}\\ {\dot{p}}_{i}=-k_{pi}p_{i}-k_{di}m_{i} \end{array}$$

where *i* sequence is *lacl, tetR, cl* and *j* sequence is *cl, lacl, tetR, k_mi_* = 1, *k_pi_* = 1, *n* = 2 and α is the control parameter. With parameters chosen as α_*i*_ = 2.5, the genetic network has a globally exponential equilibrium at $m_{i}^{*}=p_{i}^{*}=1.1150,$ which is the steady state. Evolution trajectories of *m*RNA and protein concentration levels are shown in figure 2.

**Figure 3:**
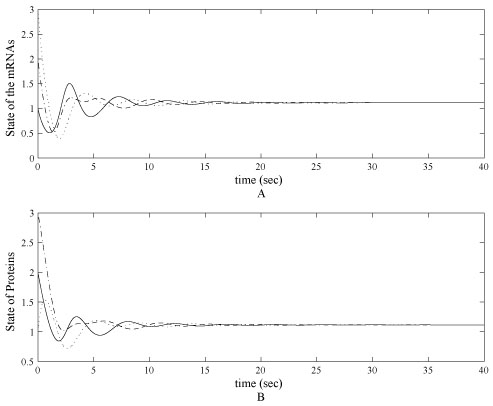
**Controlled state of mRNA and protein levels in a noise-free case**. (A) Controlled state of mRNAs of *lacl, tetR, cl* in the tri-gene regulatory in a noise-free case. The initial states of computer simulation start from initial status as [3, 2, 1]. (B) Controlled proteins *cl, lacl, tetR* in the tri-gene regulatory in a noise-free case. All states in (A) and (B) converge to 1.115 less than 20 second simulation time.

**Figure 4:**
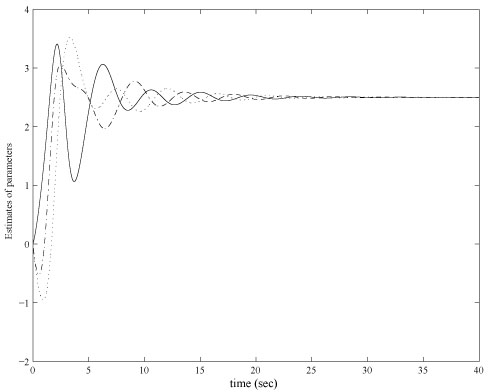
**Control parameters in a noise-free case**. Online estimated control variables α_*i*_. Initial values of α_*i*_ are all chosen as zeros without losing generality. Control parameters converge to 2.5 with in 20 second simulation time.

The control objective is to alter the current state *x* = [3, 2, 1, 1, 3, 2]^*T*^ to the desired states x^*^ by adjusting transcription rates α_i_. Assume there is no regulation at the initial state, the control law is chosen to be ${\dot{\alpha }}_{i}=-10e_{i}g_{j}.$ Evolution trajectories of *m_i_* and *p_i_* with control design are shown in figure 3. The control variables are shown in figure 4. It can be seen that the steady states converge to x^*^ and the transcription rates converge to the desired value 2.5 in the simulation. The simulation also shows that the convergence rate of the state and the parameter with the proposed control is faster than the original genetic network.

**Figure 5:**
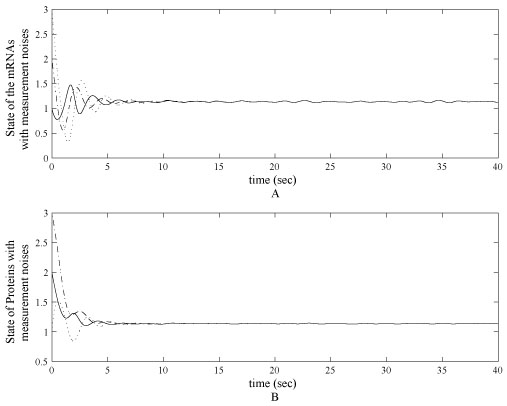
**Controlled state of mRNA and protein levels with measurement noises**. (A) Controlled state of mRNAs of *lacl, tetR, cl* in the tri-gene regulatory with measurement noises. The initial states of computer simulation start from initial status as [3, 2, 1]. (B) Controlled proteins *cl, lacl, tetR* in the tri-gene regulatory with measurement noises. The initial states of computer simulation start from initial status as [1, 3, 2]^*T*^. All states in (A) and (B) converge to 1.115 with limited error.

**Figure 6:**
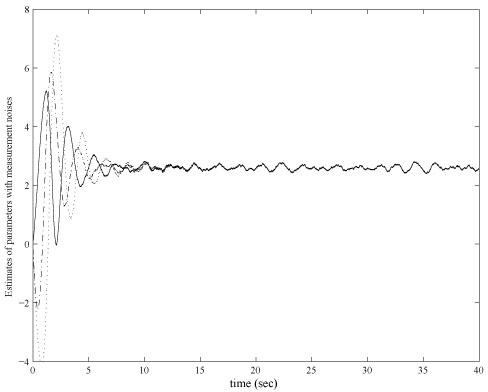
**Control parameters with measurement noises.** Online estimated control variables α_*i*_ with measurement noises. Initial values of α_*i*_ are all chosen as zeros without losing generality. Control parameters approach to 2.5 with limited error.

In order to show the robust of the control scheme, an additive white noise is injected to the measurements of the mRNA and protein concentration levels. Magnitude of the noises are bounded by 0.5. All parameter settings are the same as the ideal case illustrated above. Simulation results of the regulated mRNA and protein concentration levels are shown in figure 5 and the control of the transcription rates are shown in figure 6.

## Conclusions and future research

In the current study, a general regulatory network is presented and a new control scheme is proposed. This scheme controls the current states of the genetic network to desired values. We have obtained global convergence of the tracking error by online adjustment of the transcription rates. We have applied Lyapunov argument to the convergence analysis of the states and boundedness of the transcription rates. A tri-gene regulatory is simulated with the proposed algorithm. Effectiveness of the proposed controller design is verified by Matlab simulation for noise-free measurement and bounded noises.

In this research, interaction between the proteins and mRNAs are described by Hill functions with SUM logic. More detailed studies need to be carried out to determine the structure of a real GRN and extend the control scheme to more general cases. In addition, the estimation algorithms are developed with continuous measurers of the state. In real biological systems, such measures will be collected at specific time points. Estimation error caused by sampling effects should also be considered. Together, our results demonstrated that GRN could be regulated by artificially changing the transcription rates, to approach the desired gene expression levels.

## Competing interests

The authors declare that they have no competing interests.

## Authors' contributions

This study is carried out by the authors together. Dr. Lindsey contributes to the biology background and the discussion part. Dr. Jin contributes to the control and analysis part.
